# The Prevalence of Peripheral Erythrophagocytosis in Pediatric Immune-Mediated Hemolytic Anemia

**DOI:** 10.3390/hematolrep17010004

**Published:** 2025-01-20

**Authors:** Anselm Chi-wai Lee

**Affiliations:** Children’s Haematology and Cancer Centre, Mount Elizabeth Hospital, Singapore 228510, Singapore; anselm.cw.lee@gmail.com; Tel: +852-29171200; Fax: +852-28927599

**Keywords:** autoimmune hemolytic anemia, cold agglutinins, direct antiglobulin test, Mycoplasma pneumoniae, peripheral erythrophagocytosis, venetoclax

## Abstract

Background: Peripheral erythrophagocytosis appears to be a unique sign of acquired immune-mediated hemolytic anemia. It is said to be rare but its prevalence among patients with autoimmune hemolytic anemia has not been studied. Methods: In this retrospective study from July 2014 to June 2024, the clinical and laboratory features, treatment and outcomes of children diagnosed with autoimmune hemolytic anemia were described. The prevalence of peripheral erythrophagocytosis was compared to a group of children with hereditary spherocytosis at the time of first diagnosis seen in the same period. Results: Twelve consecutive children with autoimmune hemolytic anemia were included. There were four female patients. The mean age was 6.7 (range 0.8 to 16.6) years. The mean hemoglobin was 6.0 (range 2.5 to 8.1) g/dL. Seven patients were positive by a direct antiglobulin test, three were positive with cold agglutinins and two were positive on both tests. In seven cases, an acute infection appeared to be the precipitating factor. Mycoplasma pneumoniae infection was documented in three and suspected in another two cases. Peripheral erythrophagocytosis was present in five cases (42%) but was not found at diagnosis in any of the 16 cases of hereditary spherocytosis (*p* = 0.0081). Six children had pre-existing diseases, including two with hereditary hemolytic anemia. Conclusions: Peripheral erythrophagocytosis is a relatively common and characteristic finding in pediatric autoimmune hemolytic anemia and should be actively looked for in the evaluation of acute hemolysis, including in children with pre-existing hereditary hemolytic disorders.

## 1. Introduction

Autoimmune or immune-mediated hemolytic anemia (AIHA) is rare in pediatrics, with an estimated incidence of 0.2 per million persons younger than 20 years annually [1]. The development of antibodies targeting various red cell antigens leads to hemolysis either intravascularly or extravascularly. These antibodies are classified into warm-type and cold-type depending on the optimal temperature at which they bind to the red cells [2]. AIHA can be further categorized according to etiology. Primary AIHA takes place when there are no obvious antecedent events. The majority of childhood AIHA are secondary in origin, as a complication occurring in the course of infections, autoimmune disorders and drug exposures [1,2]. Occasionally, AIHA may be the first manifestation of the precipitating diseases [3]. When AIHA occurs with immune thrombocytopenia with or without neutropenia, the condition is better known as Evans syndrome [4].

The clinical signs and symptoms of AIHA are often non-specific and its diagnosis has to rely on laboratory findings. The hallmark of hemolysis can be seen in the peripheral blood smear. The anemia is accompanied by anisopoikilocytic red cells, polychromasia, spherocytosis, mild fragmentation and nucleated red cells. The diagnosis of immune-mediated hemolytic anemia is confirmed when warm-type antibodies (with a direct antiglobulin test) or cold-type antibodies (cold agglutinins, Donath–Landsteiner antibody) can be demonstrated [1,2].

Although the microscopic findings, especially the presence of microspherocytes, are fairly characteristic for immune-mediated hemolytic anemia, similar features can be seen in anemias associated with hereditary spherocytosis and severe thermal injuries [5]. The presence of phagocytosed red cells in the peripheral monocytes and/or neutrophils (peripheral erythrophagocytosis) appears to be a characteristic cytological feature that can distinguish immune-mediated hemolysis from the other causes of hemolytic anemia. The recognition of these morphologic findings on the blood smear provides a rapid, yet simple way of making a provisional diagnosis of autoimmune hemolytic anemia universally. The phagocytosis of red cells alone should be distinguished from multilineage phagocytosis, which is often a manifestation of the systemic inflammatory response syndrome [6]. The prevalence of peripheral erythrophagocytosis among patients with autoimmune hemolytic anemia is unknown as there are only case reports published in the literature [7,8,9,10,11,12,13,14,15,16,17,18]. This prompted the following study to answer this question from a series of pediatric cases in a single institution.

## 2. Materials and Methods

This is a retrospective chart review from the setting of a private pediatric hematology and oncology practice in Singapore. The practice receives both local and overseas patients, and referrals from other pediatric specialists. Patients are seen either as outpatient or inpatient as determined by their clinical needs.

Children 18 years old or younger who had been diagnosed and followed up for AIHA, with or without Evans syndrome, from July 2014 to June 2024 are included in the study. AIHA is defined as (1) the acute onset of anemia (hemoglobin < 10 g/dL), (2) with clinical signs or symptoms and laboratory features of hemolysis and (3) positive results on a direct antiglobulin test (DAT) and/or cold agglutinin test. The test for Donath–Landsteiner antibodies is not available in our laboratory.

At first presentation, a comprehensive medical history was obtained. The patients’ predispositions to nutritional and inherited anemias were explored. On a physical examination, the presence of jaundice was often the first clue to an underlying hemolysis. The manifestations associated with infections and immune disorders were sought. The initial laboratory investigations included a complete blood count, peripheral blood smear, renal and liver profiles and infectious diseases work-up for those presenting with fever and/or other signs of infection. Subsequent tests for reticulocyte count, LDH, antinuclear antibody and a screening for immunodeficiency were ordered if deemed necessary. Of note, all the peripheral blood films were examined by a pediatric hematologist and erythrophagocytosis was searched for in all of the cases.

The demographic data, laboratory findings, presence of peripheral erythrophagocytosis, pre-existing diseases, probable predispositions, treatment and outcomes are described. For comparison, a cohort of children with hereditary spherocytosis diagnosed during the same period is also retrieved to examine if peripheral erythrophagocytosis is seen in other types of hemolytic anemia. They all had the peripheral blood film examined by the same pediatric hematologist at the time of presentation. Hereditary spherocytosis is chosen because the condition resembles AIHA by clinical features, laboratory findings and in cytomorphology. In our practice, the diagnosis of hereditary spherocytosis is made based on the characteristic hematologic findings, negative direct antiglobulin test, and in combination with either a positive eosin-5-maleimide binding result on flow cytometry or a positive family history of hereditary spherocytosis in a first-degree relative [19]. Fisher’s exact test is used when non-parametric variables are compared. A *p* value < 0.05 is considered significant.

## 3. Results

**Figure 1:**
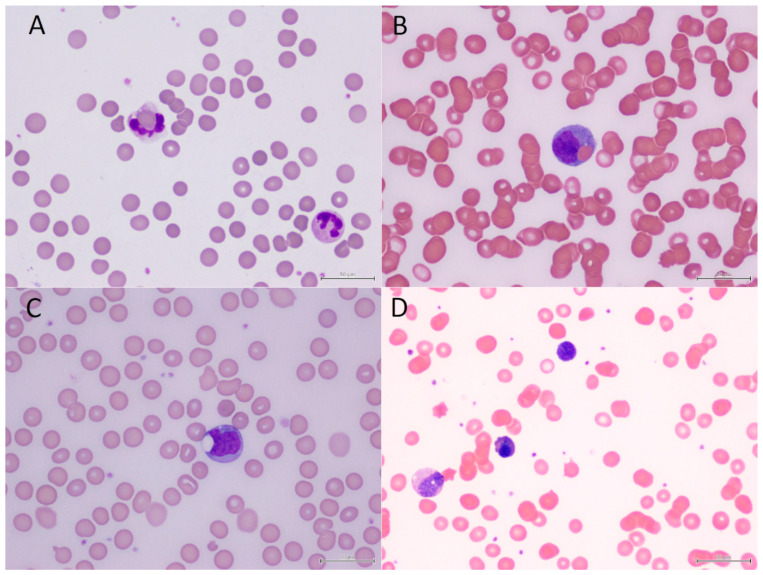
Photomicrographs of the peripheral blood smears (Wright’s; ×1000). (**A**) A six-year-old male (Case 10) born with glucose-6-phosphate dehydrogenase deficiency was admitted with Mycoplasma pneumoniae pneumonia and acute massive hemolysis. Note the spherocytes and red cell agglutination beside the neutrophilic erythrophagocytosis. (**B**) A twelve-year-old male (Case 11) with refractory acute myeloid leukemia on venetoclax therapy. Note the erythrophagocytosis in a monocyte. (**C**) A nine-month-old female (Case 12) with chronic neutropenia presenting with Mycoplasma pneumoniae pneumonia and dropping hemoglobin. The presence of spherocytosis noted in the peripheral blood was the first hint of autoimmune hemolytic anemia. Note the large vacuole with remnant erythrocytic components in the monocyte. (**D**) A five-year-old male (Case 4) with pre-existing hereditary spherocytosis presenting with Mycoplasma pneumoniae upper respiratory infection and hemolytic crisis. Spherocytosis and red cell agglutination on the blood smear led to the diagnosis of cold agglutinin-mediated hemolytic anemia.

Twelve children with a mean age of 6.7 (range 0.8 to 16.6) years were diagnosed with AIHA in the study period (Table 1). Four (33%) of them were female. Seven (58%) were positive on a DAT, three (25%) were positive for cold agglutinins and two (17%) were positive on both tests. Elevated bilirubin levels were seen in six (55%) of the eleven cases tested. Elevated AST levels were seen in five (45%) of the eleven cases tested. Elevated LDH levels were seen in three (60%) of the five cases tested. Circulating normoblasts were present in all cases. Peripheral erythrophagocytosis was detected in five (42%) cases (Figure 1A–C). Three of them (Cases 7 to 9) have been reported previously [20,21].

Half of 12 patients had pre-existing medical conditions. There were three cases of malignancy (acute myeloid leukemia, myelodysplastic syndrome and neuroblastoma, respectively) and three cases of benign hematologic diseases (chronic neutropenia, glucose-6-phosphate dehydrogenase (G6PD) deficiency and hereditary spherocytosis, respectively). None of these conditions were thought to be directly related to the occurrence of AIHA.

Eleven (92%) children were found to have a predisposing condition to the occurrence of AIHA. Infections were detected in seven (58%) cases. This included pneumonia associated with Mycoplasma pneumoniae (2 cases), upper respiratory infection associated with Mycoplasma pneumoniae (1 case), pneumonia associated with the human rhinovirus/enterovirus infection (1 case), Epstein–Barr virus-associated infectious mononucleosis (1 cases) and pneumonia presumed to be a Mycoplasma-associated illness because of cold agglutinin positivity (2 cases).

AIHA occurred in two children as the first manifestation of an underlying immune disorder. An 8-year-old female had AIHA as part of the Evans syndrome and further testing confirmed the diagnosis of systemic lupus erythematosus. A one-year-old male also had atopic eczema, thrombocytopenia and immunodeficiency. Wiskott–Aldrich syndrome was then confirmed by genetic testing [21].

In the other two children, AIHA was considered secondary to medical therapy. A 3-year-old female with pre-existing neuroblastoma received an allogeneic hematopoietic stem cell transplantation and successfully engrafted with 100% donor chimerism on Day +9 post-transplant. AIHA occurred on Day +20. A 12-year-old male who had refractory acute myeloid leukemia received palliative treatment with azacitidine and venetoclax. AIHA occurred one month afterwards and venetoclax was considered to be the culprit [22].

Two patients were transferred to other institutions for treatment. The child with refractory acute myeloid leukemia died from the leukemia shortly after the diagnosis of AIHA. The remaining nine children received treatment with intravenous immunoglobulin (4 cases), corticosteroid therapy (3 cases) and antibiotic alone (2 cases). Eight of them responded to the treatment and remained in complete remission of the AIHA at a median of 3.7 years (range 2 months to 8 years) after discharge. The child with Wiskott–Aldrich syndrome required regular treatment with intravenous immunoglobulin and was still pending for allogeneic stem cell transplantation at the time of writing.

For comparison, during the same study period, 16 cases of hereditary spherocytosis were diagnosed. None of them had peripheral erythrophagocytosis at the time of diagnosis (*p* = 0.0081).

## 4. Discussion

Autoimmune hemolytic anemia remains an uncommon diagnosis in pediatric hematology. Earlier reports suggested an annual incidence of 0.2 per million persons younger than 20 years [1,2]. A recent study from France reckoned that AIHA occurred at a rate of 0.81 per 100,000 children under 18 years old [23]. In our experience, AIHA remains an uncommon differential diagnosis among children presenting with hemolytic anemia. More cases of hereditary spherocytosis were diagnosed during the same period of time. Nevertheless, the hematomorphological features are fairly characteristic [24] and the suspicion of AIHA is often raised upon the first examination of the peripheral blood smear. The prognosis of AIHA in this series of patients is generally favorable, with the great majority of children recovering after treatment.

While the patient characteristics, treatment and outcomes from this series are similar to other reported series [25,26], two notes are of special interests to the pediatric hematologists. First, AIHA can occur in children with pre-existing hereditary hemolytic anemias. When the 5-year-old male with hereditary spherocytosis was admitted with anemia (Hb 5.1 g/dL), jaundice (bilirubin 26 µmol/L) and red cell agglutination on the blood film were noted (Figure 1D). The subsequent tests for cold agglutinin and Mycoplasma IgM were positive. He recovered after a red cell transfusion and treatment with clarithromycin. In another incident, a 6-year-old male with G6PD deficiency was admitted with pneumonia and signs of massive hemolysis (hemoglobin 6 g/dL). His parents thought it was drug-induced hemolysis. But the blood smear revealed an abundance of spherocytes, red cell agglutination and erythrophagocytosis while bite cells and ghost cells were not seen (Figure 1A). Further tests for DAT, cold agglutinin and Mycoplasma IgM were positive. The hemolysis resolved with intravenous immunoglobulin and clarithromycin treatment. Thus, acute massive hemolysis in subjects with G6PD deficiency is not always related to G6PD deficiency. Prior reports of drug-induced hemolytic crisis in subjects with G6PD deficiency should be treated with doubt unless AIHA has been actively excluded. Indeed, a recent review suggests that many drugs reported to be associated with acute hemolytic anemia in G6PD deficient subjects are probably incidental rather than causal in nature [27]. Similarly, AIHA has been reported in a child with sickle cell disease with severe hemolysis, following allosensitization from a prior blood transfusion therapy [7].

Second, the finding of peripheral erythrophagocytosis is relatively common in our series of pediatric patients with AIHA, noted in five of the twelve cases. However, none of the erythrophagocytosis was reported by the laboratory technicians. In our institution, laboratory technicians reading blood films were not aware of peripheral erythrophagocytosis, and illustrations are not available in some reference atlases [24,28]. Instead, the findings were picked up by the pediatric hematologist who routinely examined all of the blood smears in children presenting with anemia. This finding was not seen in other types of childhood hemolytic anemias in our practice. In a 2001 correspondence [29], Garratty commented that peripheral erythrophagocytosis was first noted in 1891 and had since been reported repeatedly in the intervening years. He quoted a review article by Heddle who observed that 80% of transient childhood paroxysmal cold hemoglobinuria manifested peripheral erythrophagocytosis in her compilation of cases. The phenomenon, however, was under-reported because many of those reporting on blood smears were not aware of its existence.

Without specification, the term hemophagocytosis refers to a trilineage hemophagocytosis of which all formed elements of the blood may be engulfed by the phagocytes. Trilineage hemophagocytosis is generally observed in the monocyte–macrophage system and is best exemplified in hemophagocytic lymphohistiocytosis and the macrophage activation syndrome. The pathogenesis involves a dysfunctional immune response triggered by defects in the innate immune system, impaired pathogen killing and a cytokine storm [30,31]. While hemophagocytosis is usually observed in the parenchymal organs such as the liver, spleen and bone marrow, its spillage into the peripheral blood is not uncommon [20]. Peripheral trilineage hemophagocytosis was also observed in patients admitted to the intensive care unit with systemic inflammatory response syndrome and/or hemophagocytic syndrome [6,32]. In contrast, the cellular and cytokine mechanisms behind erythrophagocytosis in AIHA is still obscure, but the involvement of a single lineage of blood cells and the observation of phagocytizing neutrophils suggest a different pathogenesis distinct from hemophagocytic lymphohistiocytosis or the systemic inflammatory response syndrome. Further research into this area will be interesting.

The main limit of this study is the small sample size and the predominantly infectious etiology in the pathogenesis of the AIHA. The unavailability of the Donath–Landsteiner antibody test and the absence of positive results on a direct antiglobulin test might have limited the diagnostic accuracy. Those who develop Evans syndrome or whose AIHA is part of a polyautoimmunity, like systemic lupus erythematosus, tend to have more chronic illnesses and adverse outcomes [4]. However, a better awareness of the presence of peripheral erythrophagocytosis will improve the diagnostic accuracy and rapidity. When more observations with larger patient cohorts can be made in a systematic manner, it may even be possible to inform if neutrophilic erythrophagocytosis, as opposed to monocytic erythrophagocyotosis alone, is a telltale feature of cold agglutinin-associated AIHA and paroxysmal cold hemoglobinuria [8,9]. Nevertheless, the finding of peripheral erythrophagocytosis does not replace the standard tests for autoimmune hemolytic anemia or red cell membrane disorders.

## 5. Conclusions

Clinical and laboratory evaluations in children suspected of autoimmune hemolytic anemia can be challenging as concurrent medical conditions are common. The finding of peripheral erythrophagocytosis may have been under-recognized and the medical personnel responsible for reading blood smears should be adequately trained. Its presence is a handy and useful clue to the diagnosis and should be routinely looked for in anemic cases. A pre-existing hereditary hemolytic anemia does not exclude the diagnosis of autoimmune hemolysis.

## Figures and Tables

**Table 1:** Clinical and laboratory features of the children with autoimmune hemolytic anemia.

Case	Sex/Age	Pre-Existing Conditions	Concurrent Diagnoses	Hb	Bilirubin	DAT	Cold Aggl.	ANA	Remarks
Absence of peripheral erythrophagocytosis									
1	M/16.6	MDS	Pneumonia	6.5	8	-	+ 1:256	-	Responded to prednisolone treatment
2	F/2.5	Nil	Pneumonia	5.9	6	-	+ 1:1024	ND	Resolved after transfusion and antibiotic treatment
3	F/8.6	Nil	SLE/Evans syndrome	8.1	32	+ (poly)	+ 1:512	+	-
4	M/5.4	HS	URI, Mycoplasma infection	5.1	26	-	+ 1:64	ND	Resolved after transfusion and antibiotic treatment
5	F/5.9	Nil	EBV-IM	5.6	11	+ (poly)	ND	-	Resolved after transfusion and corticosteroid treatment
6	M/15.1	Nil	Nil	2.5	ND	+ (IgG and C3)	ND	-	Resolved after transfusion and corticosteroid treatment
7	M/1.2	Nil	WAS, EBV and CMV co-infection	6.6	22	+ (IgG)	ND	ND	Partial response after IVIG
Presence of peripheral erythrophagocytosis									
8	M/2.5	Nil	Pneumonia, hRV/EV infection	4.8	69	+ (poly)	-	+	Resolved after transfusion and single dose of IVIG
9	F/3.8	Neuroblastoma	HSCT	6.1	30	+ (poly)	ND	ND	Resolved after transfusion and single dose of IVIG
10	M/6.2	G6PD deficiency	Pneumonia, Mycoplasma infection	7.8	24	+ (poly)	+ 1:64	ND	Resolved after single dose of IVIG
11	M/12.4	AML	Venetoclax therapy	7.3	40	+ (IgG)	ND	+	Succumbed from refractory leukemia
12	M/0.8	Chronic neutropenia	Pneumonia, Mycoplasma infection	7.9	3	+ (poly)	ND	-	

Abbreviations: AML—acute myeloid leukemia; ANA—antinuclear antibody; bilirubin (normal < 22 μmol/L); CMV—cytomegalovirus; cold aggl.—cold agglutinin; DAT—direct anti-globulin test; EBV—Epstein–Barr virus; F—female; G6PD—glucose-6-phosphate dehydrogenase; Hb—hemoglobin (g/dL); hRV/EV—human rhinovirus/enterovirus; HS—hereditary spherocytosis; HSCT—hematopoietic stem cell transplantation; IM—infectious mononucleosis; IVIG—intravenous immunoglobulin; M—male; MDS—myelodysplastic syndrome; ND—not done; neg—negative; SLE—systemic lupus erythematosus; URI—upper respiratory treatment; WAS—Wiskott–Aldrich syndrome.

## Data Availability

The data presented in this study are available upon request from the corresponding author.
